# Supplementary material for: Pain Relieving Effect of-NSAIDs-CAIs Hybrid Molecules: Systemic and Intra-Articular Treatments against Rheumatoid Arthritis
Source: Int J Mol Sci. 2019 Apr 18;20(8):1923. doi: 10.3390/ijms20081923 (PMC6514875; doi:10.3390/ijms20081923)
Supplement: Supplementary file 1 [file ijms-20-01923-s001.pdf]

Pain relieving effect of new NSAIDs-CAIs hybrid molecules: systemic and intra-articular treatments against rheumatoid arthritis

Laura Micheli, Murat Bozdag, Ozlem Akgul, Fabrizio Carta, Clizia Guccione, Maria Camilla Bergonzi, Annarita Bilia, Lorenzo Cinci, Elena Lucarini, Carmen Parisio, Claudiu T. Supuran, Carla Ghelardini, Lorenzo Di Cesare Mannelli

Corresponding author: Lorenzo Di Cesare Mannelli, Dept. of Neuroscience, Psychology, Drug Research and Child Health - NEUROFARBA - Pharmacology and Toxicology Section, University of Florence, Viale Gaetano Pieraccini 6, 50139, Florence, Italy.
Phone: +39-055-2758395
E-mail: lorenzo.mannelli@unifi.it

Supplementary materials

*General procedure for synthesis of compounds **4** and **5**.*

Nuclear magnetic resonance (1H NMR, 13C NMR, and 19F-NMR) spectra were recorded using a Bruker Advance III 400 MHz spectrometer using DMSO-d6 as solvent. The chemical shifts are reported in parts per million (ppm), and the coupling constants (J) are expressed in Hertz (Hz). The splitting patterns are designated as s, singlet; d, doublet; t, triplet; q, quartet; m, multiplet; brs, broad singlet; and dd, doublet of doublets. The correct assignment of exchangeable protons (i.e., OH and NH) was carried out by means of the addition of D2O. Analytical thin-layer chromatography (TLC) was done on Merck silica gel F-254 plates. Flash chromatography was performed on Merck silica gel 60 (230–400 mesh ASTM) as the stationary phase, and appropriate mixtures of ethyl acetate/n-hexane were the eluents. Melting points (m.p.) were measured in open capillary tubes with a Gallenkamp MPD350.BM3.5 apparatus and are uncorrected. The HPLC was performed by using a Waters 2690 separation module coupled with a photodiode array detector (PDA Waters 996) using a Nova-Pak C18 4 μm 3.9 mm × 150 mm (Waters) silica-based reverse phase column. The sample was dissolved in 10% acetonitrile/H2O and an injection volume of 45 μL. The mobile phase (flow rate 1.0 mL/min) was a gradient of H2O + trifluoroacetic acid (TFA) 0.1% (A) and acetonitrile + TFA 0.1% (B), with steps as follows: (A%:B%), 0–10 min 90:10, 10–25 min gradient to 60:40, 26:28 min isocratic 20:80, and 29–35 min isocratic 90:10. TFA (0.1%)

in water as well in acetonitrile was used as the counterion. All compounds reported here were ≥95% HPLC pure.

Complete Freund's adjuvant-induced rheumatoid arthritis

Briefly, the rats were lightly anesthetized by 2% isoflurane, the left leg skin was sterilized with 75% ethyl alcohol and the lateral malleolus located by palpation. A 28-gauge needle was then inserted vertically to penetrate the skin and turned distally for insertion into the articular cavity at the gap between the tibiofibular and tarsal bone until a distinct loss of resistance was felt. A volume of 50 µl of CFA was then injected (day 1). Control rats received 50 µl of saline solution (day 1) in the tibiotarsal joint.

HPLC PDA analysis

Separation was performed at 27 °C on Agilent ZORBAX Eclipse XDB columns C18 RP (4.6 mm x 250 mm, 5µm). The mobile phase consisted of H_2O at pH 3.2 adjusted by formic acid (solvent A) and ACN (solvent B). The flow rate was 0.8 mL/min and the total run time was 32 min. The following gradient profile was used: 0 –2 min, 0-2% B; 2–25 min, 2–100% B; 25–30 min 100% B; 30–32 min, 100 -2% B, with equilibration time of 5 min.

The sample injected volume was 10 µL. UV spectra were recorded between 200 and 600 nm. Chromatographic profiles were recorded at 254 and 280 nm. The identification of the constituents was performed by comparing the retention time and the UV spectra of the peaks in the samples with those of authentic reference samples.

Preparation of liposomes

Briefly, P90G (66 mg/mL) and cholesterol (20 mg/mL) were dissolved in dichloromethane and the organic solvent were evaporated under vacuum. The dry lipid film was hydrated by addition of 10 mL of deionized water. The dispersion was mechanically stirred at 37 °C in water bath for 30 minutes. In order to reduce the dimensions of the vesicles from MLV to SUV, the liposomal dispersions were centrifuged for 5 minutes at 10000 rpm. Liposomes loaded with **4** and with **5** were prepared as described above. After solubility studies, the compound 4 was, firstly, dissolved in a mixture 70:30 Acetone: EtOH while the compound **5** was solubilized in acetone. These solutions were then added to the lipid film mixture, before the hydration.

Three concentrations of molecules were tested: 1mg/mL, 3mg/mL, 5 mg/mL in order to obtain the highest encapsulation efficiency (EE) of each loaded –vesicle.

Characterization of liposomes

Particle size was measured by a Dynamic Light Scattering (DLS), Zetasizer Nano series ZS90 (Malvern Instruments, Malvern, UK) set at 25 °C. Time correlation functions were analysed to obtain the hydrodynamic diameter of the particles (Zh) and the particle size distribution (polydispersity index, PDI) using the ALV-60X0 software V.3.X provided by Malvern. Autocorrelation functions were analysed by the cumulants method (fitting a single exponential to the correlation function to obtain the mean size (nm) and PDI.

Zeta potentials (ζ-potentials) of the liposome systems were measured using the same instrument. For all samples, an average of three measurements at a stationary level was taken. The temperature was kept constant at 25 °C by a Haake temperature controller. The ζ-potential was calculated from the electrophoretic mobility, lE, using the Henry correction to Smoluchowski's equation.

Encapsulation efficiency (EE%)

EE% is defined as the amount of drug entrapped in the vesicles in relation to the total amount of drug present during the vesicle formation and entrapment procedure. Free **4** and with **5** were removed by means of dialysis. Liposomal formulation was transferred in a dialysis bag that was stirred in 800 mL of water at room temperature for 2 h, as previously reported by Isacchi B et al. (2012). The aqueous medium was refreshed once. Purified liposomes were collected and then disrupted using the dilution method with organic solvent (methanol), then were submitted to ultrasonication in water bath for 30 minutes. After centrifugation at 12,000 rpm for 5 min, the content of loaded compounds into the liposomes were quantified by HPLC PDA analysis, using **4** and with **5** as external standards. EE% is reported as the percentage of drug entrapped in the vesicles: EE (%) = {(amount of drug entrapped in the vesicles)/(total amount of drug used in the preparation)} x 100.

Stability studies in storage conditions

Stability of both loaded liposomes was studied over 15 days. Vesicles dispersions were kept at 4 °C and at fixed intervals physical and chemical stabilities were evaluated. Physical stability was checked by DLS monitoring size and PDI. Chemical stability was checked quantifying the drug content by HPLC analysis.

Paw-pressure test

Briefly, a constantly increasing pressure was applied to a small area of the dorsal surface of the hind paw using a blunt conical probe by a mechanical device. Mechanical pressure was increased until vocalization or a withdrawal reflex occurred while rats were lightly restrained. Vocalization or withdrawal reflex thresholds were expressed in grams. Rats scoring below 40 g or over 75 g during the test before drug administration were rejected (25%). For analgesia measures, mechanical pressure application was stopped at 120 g.

Von Frey test

An electronic Von Frey hair unit (Ugo Basile, Varese, Italy) was used: the withdrawal threshold was evaluated by applying force ranging from 0 to 50 grams with a 0.2 gram accuracy. A punctuate stimulus was delivered to the mid-plantar area of each posterior paw from below the meshy floor through a plastic tip and the withdrawal threshold was automatically displayed on the screen. The paw sensitivity threshold was defined as the minimum force required to elicit a robust and immediate withdrawal reflex of the paw. Voluntary movements associated with locomotion were not considered as a withdrawal response. Stimuli were applied on each posterior paw with an interval of 5 seconds. Measurements were repeated five times and the final value was obtained by averaging the five measurements (Di Cesare Mannelli et al., 2013; Sakuraki et al., 2009).

Beam Balance test

A rectangular beam (3.2 cm wide, 122 cm long and 63.5 cm tall) was suspended between two tables (105 cm tall for the top of the beam). A black box is placed at the end of the of the beam as the finish point. Animals were placed perpendicularly on the midpoint of the beam and allowed to traverse the beam for 120 s. A score to the motor abilities of the animal was given: 0, correct gait; 1, clings with the 4 paws; 2, slips with one paw; 3, slips with two paws; falls in a time less than 60 sec.

Supplementary Figures and Table

Supplementary Panel S1

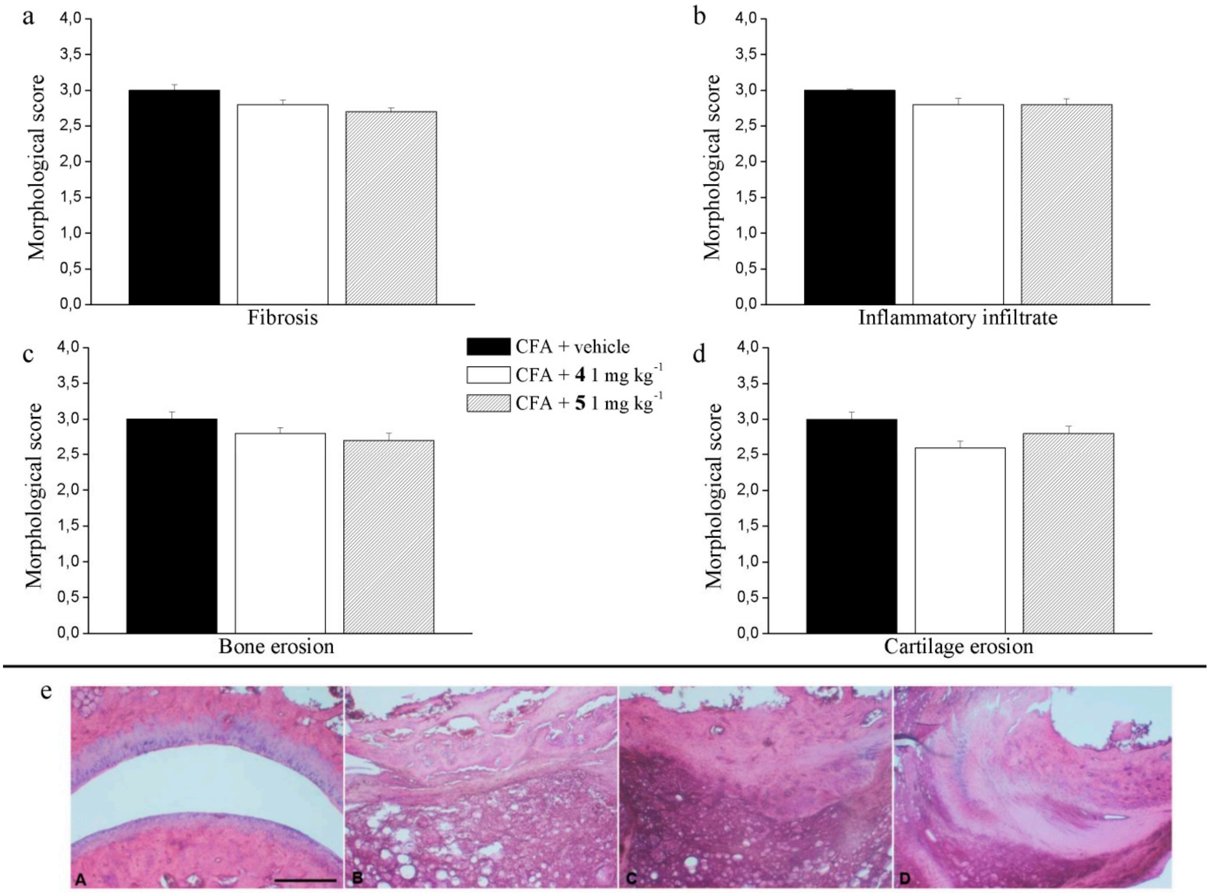

Panel S1. Histological analysis of the tibio-tarsal joint. Quantification of morphological parameters by specific score (0: absent; 1: light; 2: moderate; 3: severe): a) fibrosis; b) inflammatory infiltrate; c) bone erosion; d) cartilage erosion. Control animal had all morphological score equal to 0 and were not reported in graphs. E) Representative images of tibio-tarsal joint: a) vehicle + vehicle treated animals; b) CFA + vehicle treated animals; c) CFA + **4** 1 mg kg^{-1} treated animals; d) CFA + **5** 1 mg kg^{-1} treated animals. Scale bar 200 µm. The value represent the mean of eight rats performed in two different experimental sets.

Supplementary Table 1: Characterization of lipo 4 and lipo 5

Nanoformulation	**Size (nm)**	**PDI**	**ζ-Potential (mV)**	**EE%**
Empty liposome	162.3 ± 0.5	0.22 ± 0.01	-22.0 ± 1.8	-
**Liposome loaded with 1 mg/mL of 4**	234.2 ± 0.8	0.21 ± 0.01	-21.0 ± 2.2	29.6 ± 1.6
Liposome loaded with 3 mg/mL of **4**	185.2 ± 2.1	0.26 ± 0.20	-19.2 ± 1.2	9.3 ± 0.6
Liposome loaded with 5 mg/mL of **4**	211.5 ± 1.5	0.29 ± 0.10	-20.0 ± 0.9	6.7 ± 0.9
**Liposome loaded with 1 mg/mL of 5**	235.4 ± 0.2	0.34 ± 0.01	-22.1 ± 0.8	74.8 ± 6.3
Liposome loaded with 3 mg/mL of **5**	153.6 ± 1.2	0.22 ± 0.10	-19.3 ± 1.6	25.2 ± 2.1
Liposome loaded with 5 mg/mL of **5**	127.6 ± 1.6	0.23 ± 0.05	-18.0 ± 2.0	19.4 ± 0.6

Final characterization of empty and loaded nanoformulations in term of size (nm), homogeneity (PDI), ζ-potential (mV) and encapsulation efficiency (EE%). Data are shown as means of triplicate measurements (± SD)

Supplementary Figure S1: Physical stability of liposome loaded with 1mg/ml of **4** in term of mean diameter by DLS analysis

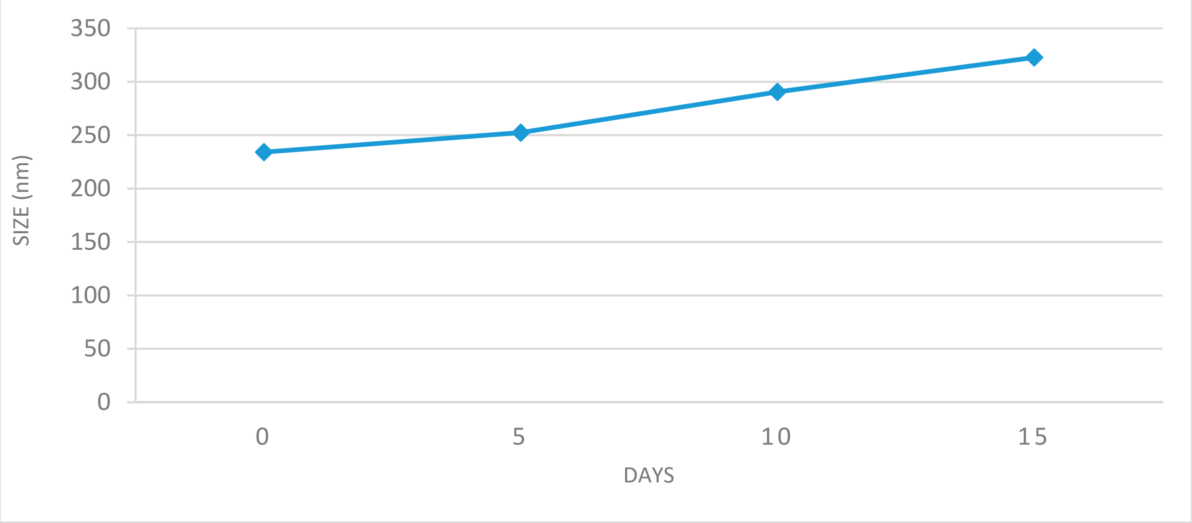

Data are show as means of triplicate.

Supplementary Figure S2: Physical stability of liposome loaded with 1mg/ml of **5** in term of mean diameter by DLS analysis.

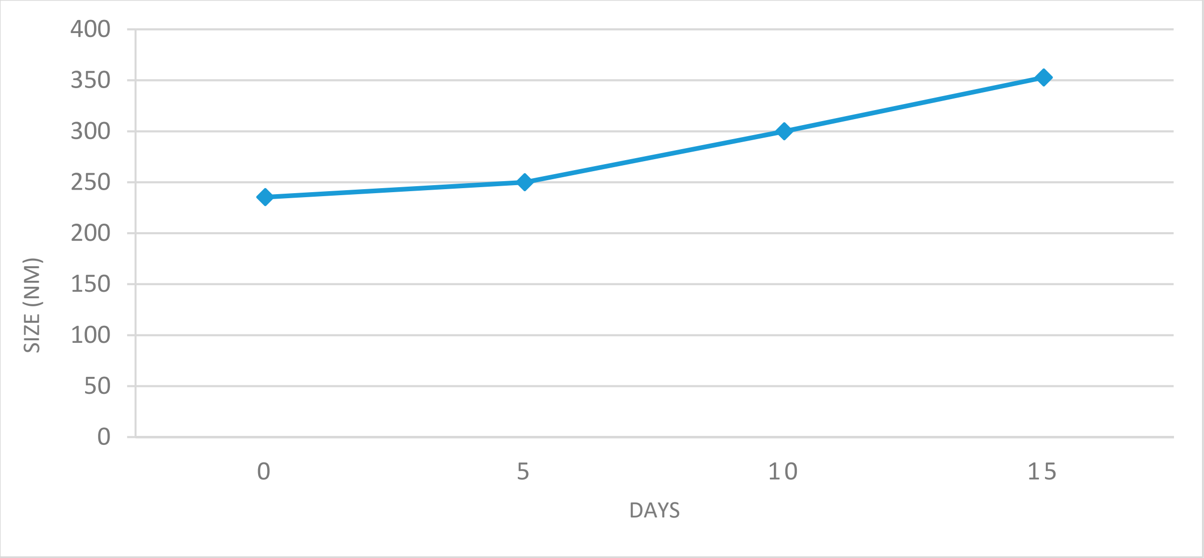

Data are show as means of triplicate

Supplementary Figure S3: Physical stability of liposome loaded with 1mg/ml of **4** in term of polydispersity (PDI) by DLS analysis

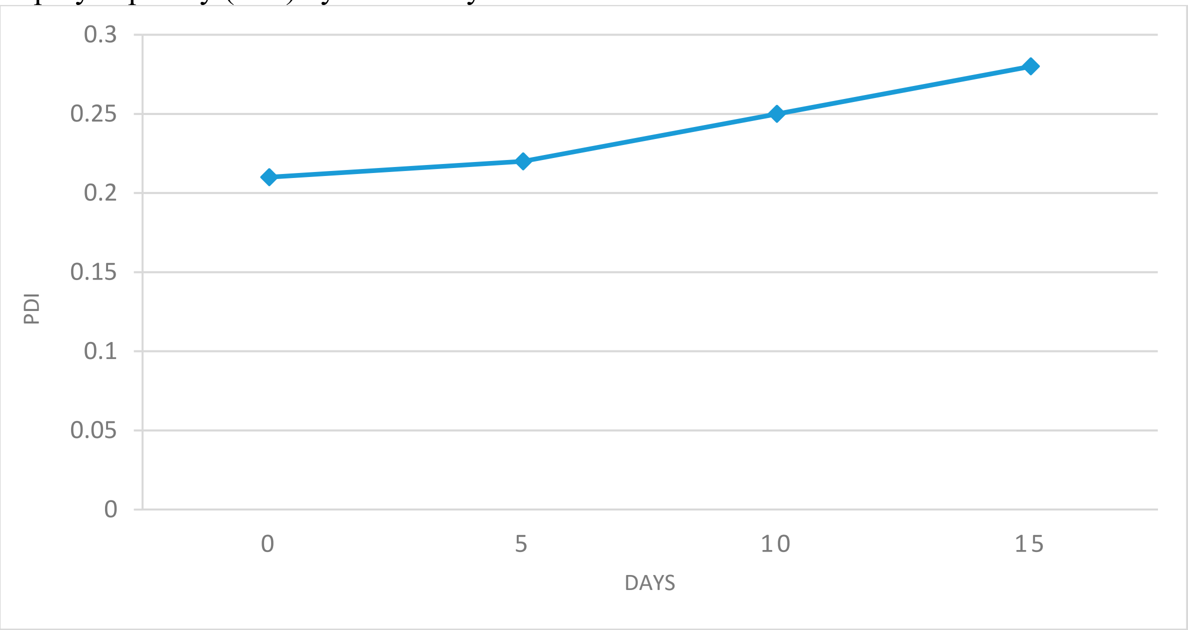

Data are show as means of triplicate.

Supplementary Figure S4: Physical stability of liposome loaded with 1mg/ml of **5** in term of polydispersity (PDI) by DLS analysis.

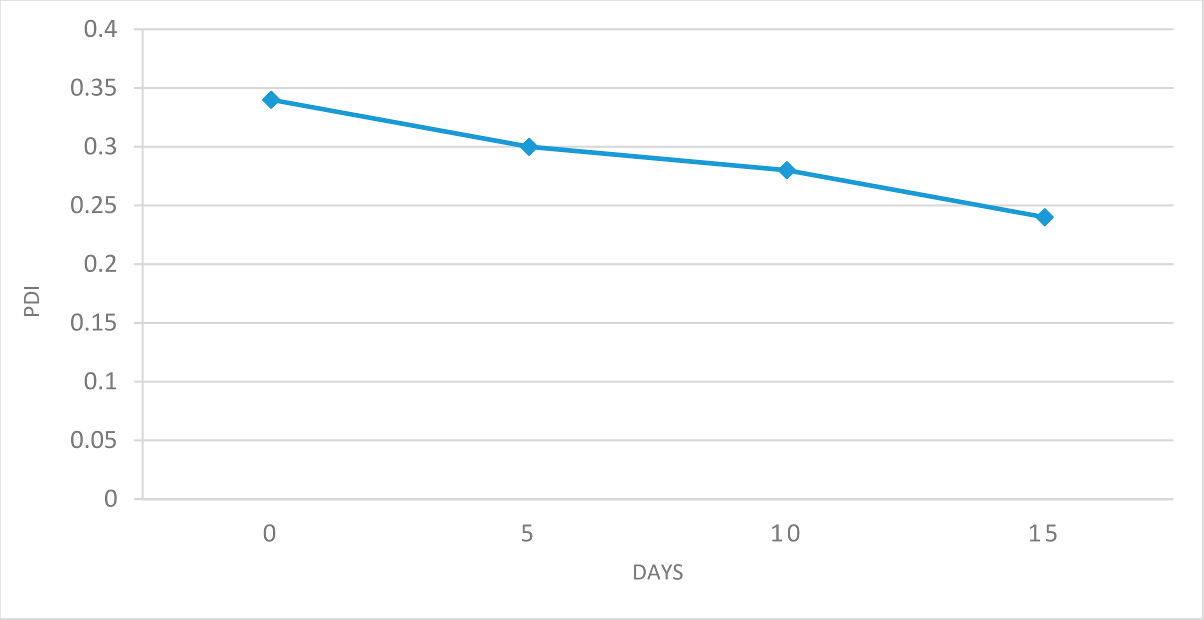

Data are show as means of triplicate.

Supplementary Figure S5: Chemical stability of liposome loaded with 1mg/ml of **4** in term of encapsulation efficacy (EE%) by HPLC PDA analysis.

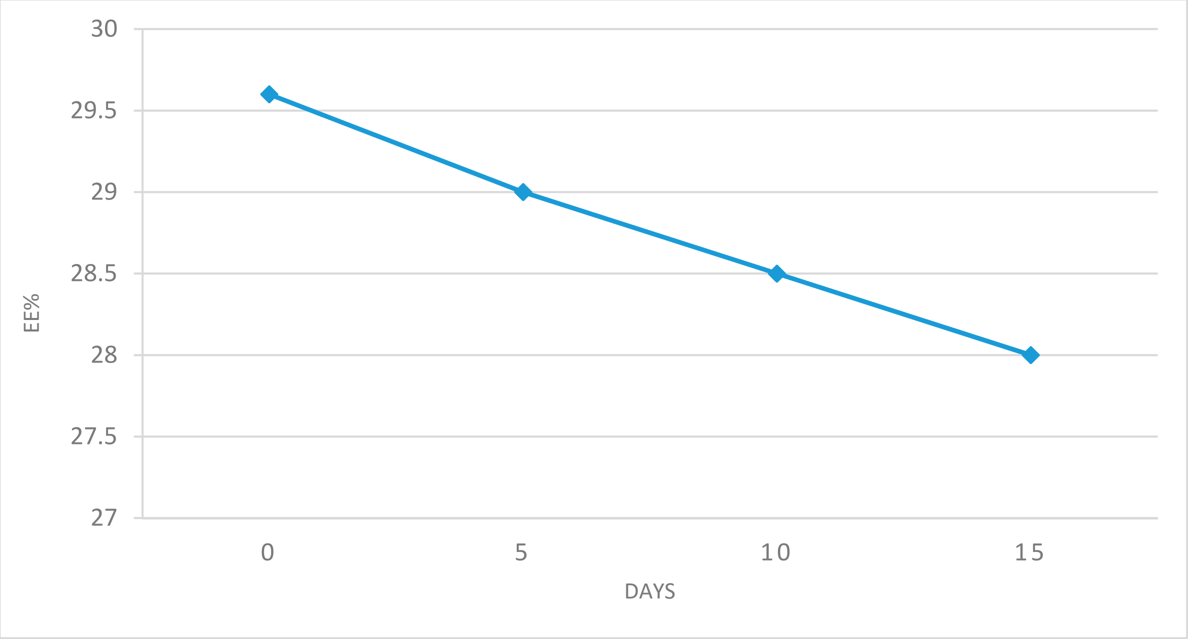

Data are show as means of triplicate.

Supplementary Figure S2: Chemical stability of liposome loaded with 1mg/ml of **5** in term of encapsulation efficacy (EE%) by HPLC PDA analysis.

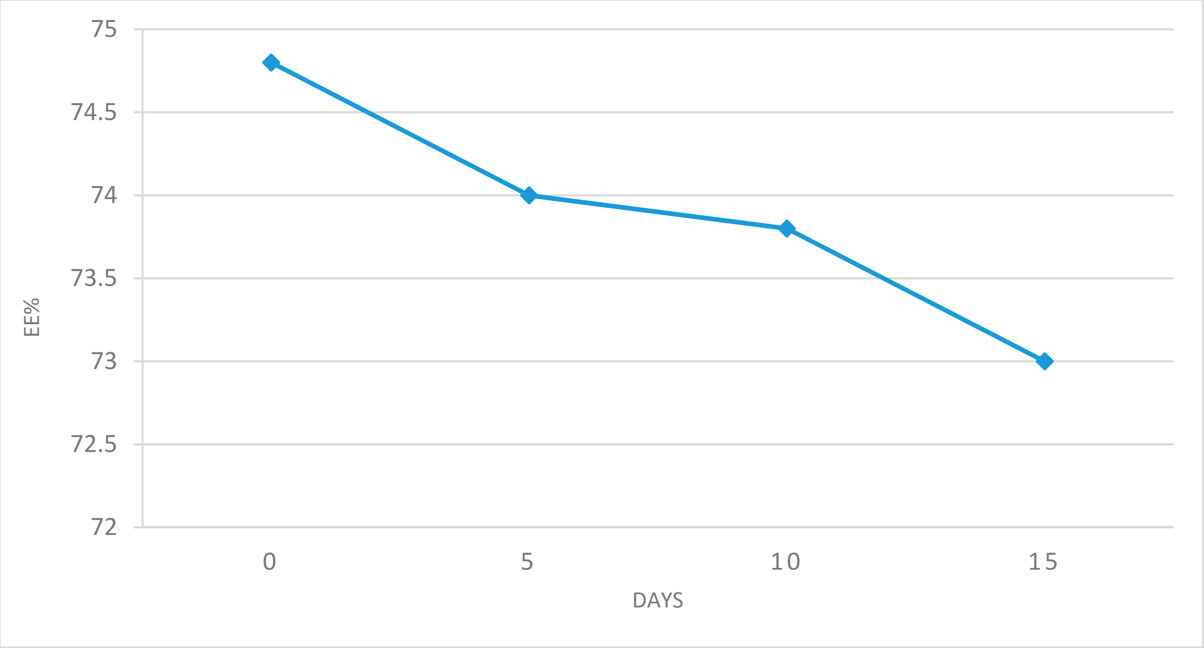

Data are show as means of triplicate.

Supplementary Panel S2

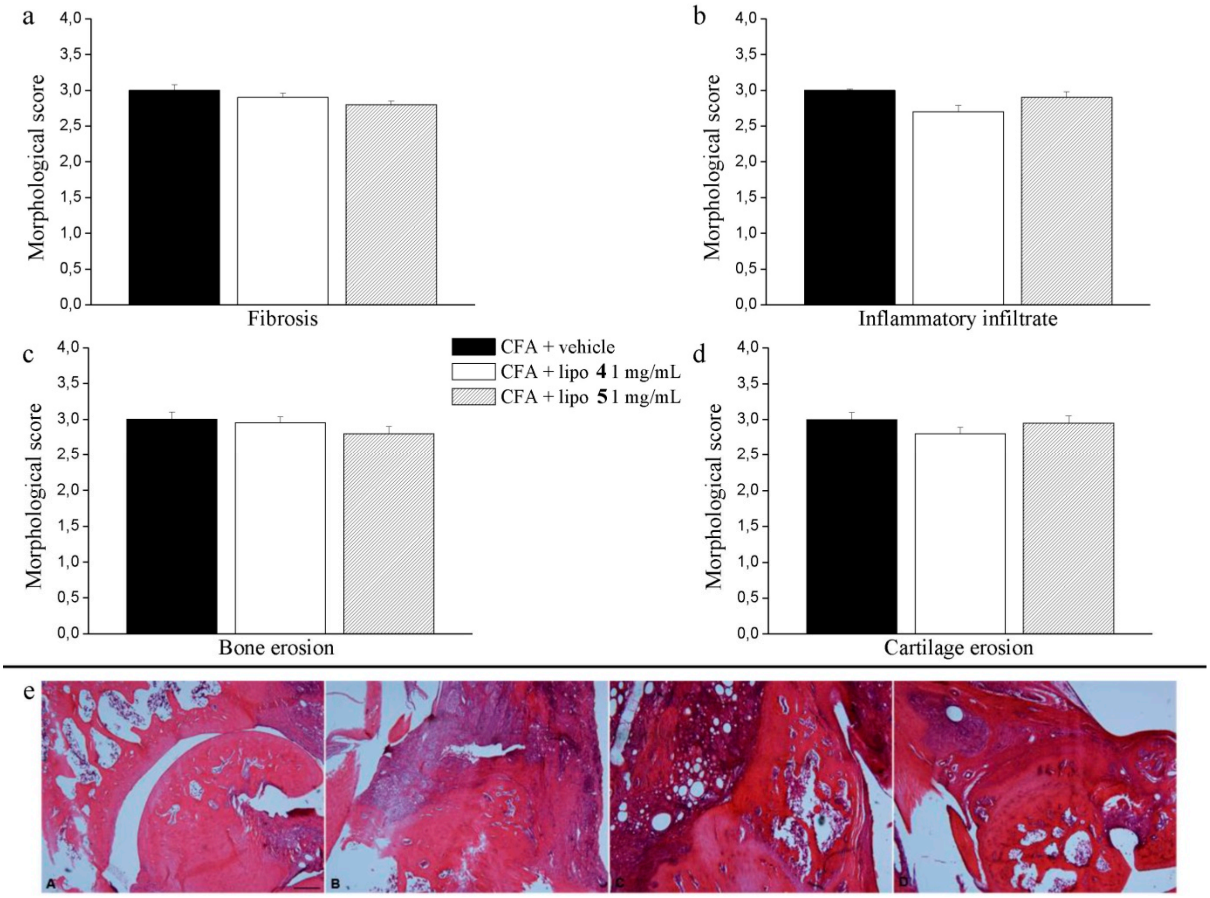

Panel S2. Histological analysis of the tibio-tarsal joint. Quantification of morphological parameters by specific score (0: absent; 1: light; 2: moderate; 3: severe): a) fibrosis; b) inflammatory infiltrate; c) bone erosion; d) cartilage erosion. Control animal had all morphological score equal to 0 and were not reported in graphs. E) Representative images of tibio-tarsal joint: a) vehicle + vehicle treated animals; b) CFA + vehicle treated animals; c) CFA + lipo **4** 1 mg kg^{-1} treated animals; d) CFA + lipo **5** 1 mg kg^{-1} treated animals. Scale bar 200 μm. The value represent the mean of eight rats performed in two different experimental sets.
